# Monitoring adolescent sexual and reproductive health

**DOI:** 10.2471/BLT.16.170688

**Published:** 2016-03-01

**Authors:** Michelle J Hindin, Özge Tunçalp, Caitlin Gerdts, Jessica D Gipson, Lale Say

**Affiliations:** a Department of Reproductive Health and Research, World Health Organization, 20 Avenue Appia, 1211 Geneva 27, Switzerland.; b Ibis Reproductive Health, San Francisco, United States of America (USA).; c Department of Community Health Sciences, University of California - Los Angeles Fielding School of Public Health, Los Angeles, USA.

The year 2016 is a critical year for adolescent sexual and reproductive health (ASRH), when two key global health strategies – the 2030 Agenda for Sustainable Development^1^ and the United Nations Global Strategy for Women’s, Children’s and Adolescents’ Health^2^ – are being put into effect. Both strategies will inform and catalyse the collaborative and global efforts on ASRH for the next 15 years. While the goals and targets for these strategies have been agreed upon, the indicators to track the targets are currently being debated. The chosen indicators will have wide-reaching implications for ASRH programming, policy-making and resource allocation at all levels: globally, nationally and locally. As conversations on indicator development continue, we must accurately define what we are and what we are not measuring and acknowledge the limitations of the chosen indicators.

The Programme of Action of the International Conference on Population and Development,^3^ adopted in 1994, highlighted the importance of addressing ASRH issues including unwanted pregnancy and unsafe abortion.^3^ This objective was partially operationalized in the Millennium Development Goals (MDGs) framework, under Goal 5 “improve maternal health” and was monitored using the indicator “adolescent birth rate” defined as “number of births per 1000 women ages 15–19 years”.^4^ However, when the aim is to reduce unwanted pregnancies among adolescents, monitoring only births – and not pregnancies – tells only part of the story. Birth rates can decrease or increase while pregnancy rates remain unchanged. Evidence suggests a worldwide decline in the adolescent birth rate, even in countries where access to effective contraception is poor.^5^ For example, in sub-Saharan Africa, an estimated 3.3 million adolescents, aged 15–19 years, have an unmet need for modern contraception,^6^ yet the adolescent birth rate in sub-Saharan Africa has declined from 150.2 per 1000 adolescent women in 1960 to 108.8 in 2013.^7^ The global decline in adolescent birth rates may be caused by a decrease in the proportion of sexually active adolescents, an increase in the proportion of adolescents using contraceptives, or an increase in the proportion of adolescents terminating pregnancies through induced abortion. In fact, existing evidence suggests that there has not been a decline in sexual activity rates among adolescents, and in some cases there has been an increase.^8^ The contraceptive use rate in this age group has been largely stable over the past 20 years.^8^ On average, in countries where abortion is more accessible, the adolescent birth rate is lower.^9^

This body of evidence highlights that solely tracking adolescent birth rates will provide insufficient data to inform country-specific interventions, policies and resource allocation for adolescent sexual and reproductive health. The reported decline in global adolescent birth rates is not fully explained by the decline in the adolescent pregnancy rate. By focusing only on birth rates, we are failing to address safe and unsafe abortion. Unsafe abortion is a major cause of maternal morbidity and mortality.^10^^,^^11^ Without accounting for abortion, we are failing to capture the adolescent pregnancies that do not result in a birth.

To address the challenge of under-reporting in accurately measuring pregnancies among adolescents, we need innovative ways to generate an estimate for adolescent pregnancy rates. One approach is to use reported rates of sexual activity and contraceptive coverage from population-based surveys, in combination with adolescent birth rates. Such innovative estimation methods have been tried using data on adolescent birth rates and legal status of abortion to estimate adolescent pregnancy rates.^12^ Another approach towards advancing our understanding of adolescent pregnancies is to find better ways to integrate questions on abortion as part of the set of reproductive health indicators collected through existing Demographic and Health Surveys and national health information systems. As sexual activity, pregnancy and abortion are likely to be underreported in face-to-face interviews, innovative methods of interviewing and documenting sensitive behaviours – such as audio-computer assisted self-interviews or confidential response sheets for specific questions – should be considered. By monitoring adolescent pregnancies and their outcomes, we can ensure resources are better allocated to meet the sexual and reproductive health needs of adolescents.

The global decline in the adolescent birth rate should remove adolescent sexual and reproductive health needs from the global agenda. Adolescents’ need for contraception and safe abortion and their right to plan pregnancies are still unfinished business for the 2030 Agenda for Sustainable Development. 
