# Corrigendum

**DOI:** 10.2471/BLT.16.120416

**Published:** 2016-04-01

In Volume 94, Issue 3, March 2016, page 159, the first line of the last paragraph should read: “The global decline in the adolescent birth rate should not remove adolescent sexual and reproductive health needs from the global agenda.”

